# Cognate RNA-Binding Modes by the Alternative-Splicing Regulator MBNL1 Inferred from Molecular Dynamics

**DOI:** 10.3390/ijms232416147

**Published:** 2022-12-18

**Authors:** Àlex L. González, Daniel Fernández-Remacha, José Ignacio Borrell, Jordi Teixidó, Roger Estrada-Tejedor

**Affiliations:** Grup de Química Farmacèutica (GQF), IQS School of Engineering, Universitat Ramon Llull, 08017 Barcelona, Spain

**Keywords:** myotonic dystrophy, molecular dynamics, MBNL1

## Abstract

The muscleblind-like protein family (MBNL) plays a prominent role in the regulation of alternative splicing. Consequently, the loss of MBNL function resulting from sequestration by RNA hairpins triggers the development of a neuromuscular disease called myotonic dystrophy (DM). Despite the sequence and structural similarities between the four zinc-finger domains that form MBNL1, recent studies have revealed that the four binding domains have differentiated splicing activity. The dynamic behaviors of MBNL1 ZnFs were simulated using conventional molecular dynamics (cMD) and steered molecular dynamics (sMD) simulations of a structural model of MBNL1 protein to provide insights into the binding selectivity of the four zinc-finger (ZnF) domains toward the GpC steps in YGCY RNA sequence. In accordance with previous studies, our results suggest that both global and local residue fluctuations on each domain have great impacts on triggering alternative splicing, indicating that local motions in RNA-binding domains could modulate their affinity and specificity. In addition, all four ZnF domains provide a distinct RNA-binding environment in terms of structural sampling and mobility that may be involved in the differentiated MBNL1 splicing events reported in the literature.

## 1. Introduction

Alternative splicing (AS) of pre-mRNA determines the expression of distinct functional protein isoforms starting from a relatively small number of genes in eukaryotes [1,2,3]. AS regulators bind to pre-mRNA molecules and modulate the expression of different protein isoforms, which allows different exon assembly combinations. Changes in RNA sequences due to AS may result in isoforms with different motions and conformations, which varies their function, and cellular localization, or it may even regulate protein levels by leading to non-productive splicing RNA turnover [4,5,6,7].

Intriguingly, studies on the sequence and structure of AS revealed that AS was primarily found in intrinsically disordered regions with highly heterogeneous structural ensembles which could sometimes involve the whole protein [4,6,8]. Given this correlation, some studies pointed out that the modulation of AS may be driven by the modification of protein structural sampling. The work of Romero et al. [4] suggested that small changes in AS sites could modify the specificity of the protein and revealed that a relationship between AS and intrinsic disorder exists. Barbany et al. [6] compared the isoforms of two AS proteins and reported that AS was not necessarily related to either local or global changes in the size of protein fluctuations. However, authors noticed that AS induced subtle changes in protein dynamics, which might explain its specificity for RNA targets, and AS might be modulated through changes in cavity couplings [9].

Muscleblind-like proteins (MBNL) are AS factors that are encoded in mammals by *MBNL1*, *MBNL2,* and *MBNL3* genes. MBNL proteins can act as either repressors or activators of splicing in several transcripts [10,11,12]. They belong to a family of tissue-specific RNA metabolism regulators that play a key role in terminal muscle differentiation. All three family members share four highly conserved zinc-finger domains (ZnF) for recognizing specific pre-mRNA and mRNA targets [11]. These RNA binding domains of the CCCH type are arranged in tandem pairs, of which ZnF1/ZnF2 are positioned toward the N-terminal region, and ZnF3/ZnF4 are in the middle part of the sequence. Each domain contains a different spacing between the zinc-coordinated residues. ZnF1 and ZnF3 contain a CX7CX6CX3H motif, whereas ZnF2 and ZnF4 contain a CX7CX4CX3H sequence. The RNA binding faces in each domain are arranged back-to-back, creating an anti-parallel alignment of RNA binding to ZnF domains [11]. Particularly, MBNL1 has been the main focus of intense studies over the past years due to its implication in the Myotonic Dystrophy (DM) pathogenic pathway [13,14,15,16,17,18,19]. Myotonic Dystrophy type 1 (DM1) is caused by the expansion of RNA CUG repeats which bind and sequester MBNL1 [19,20,21]. SELEX experiments determined that the optimal MBNL binding RNA sequence consisted of multiple YGCY consensus motifs (with Y as a pyrimidine base), which explains the binding to CUG expansions and the inactivation of its normal functions [15]. Such a binding mechanism was further confirmed by a crystallographic structure of MBNL1 that showed the interaction of the ZnF3/4 tandem with an RNA fragment (PDB id: 3D2S). The crystallographic model confirmed that Phe202 and Tyr236 aromatic residues intercalate between the GC step. Additionally, several hydrogen bonds are formed between the GC motif and the side chains in the protein [11], which may also help explain its high affinity for the YGCY motif.

The human gene *MBNL1* includes twelve exons, ten of which correspond to the coding sequence (exons 1–10), and six that undergo alternative splicing (exons 3, 5, and 6–9). Thus, there are at least seven *MBNL1* mRNA variants which lead to extensive alternative splicing regulation [22]. Analysis of *MBNL1* deletion constructs proved that exon 5 and a five amino acid region in exon 6 are essential for nuclear localization. On the contrary, exons 1, 2, and 4 encode the ZnF domains and exon 7 participates in dimerization and induces the formation of ring-like structures upon binding to RNA [22,23].

Nevertheless, relevant questions about MBNL1 binding sites’ architecture remain to be addressed. For instance, the amino acid sequences of ZnF1/2 and ZnF3/4 are very well conserved; however, despite their high structural resemblance, truncated versions of MBNL1 showed a differentiated binding affinity for target RNAs. Moreover, the deletion of either ZnF1 or ZnF4 alone greatly diminishes their interaction with CUG repeats in vivo, suggesting that ZnF1/2 and ZnF3/4 domains are not functionally equivalent [12,24]. Additionally, the linker sequence encoded by exon 3, which separates the two tandems, also determines the MBNL1-RNA interaction [11,12]. In that context, heterogeneity of the MBNL domains regarding sequence conservation, coevolution, and mobility could provide new insights into the MBNL-RNA binding interaction mechanism. Moreover, less conserved residues among CCCH regions may have a determinant impact on local and global fluctuations and on RNA binding events. In this study, we address these questions using a combination of molecular dynamics (MD) simulations and bioinformatic analyses.

## 2. Results and Discussion

### 2.1. Sequence Coevolution of the CCCH Domain

MBNL1 mainly interacts with YGCY consensus motif RNAs through its four CCCH ZnFs, although some other regions of the protein are hypothesized to contribute or play an additive role [12,22]. ZnF domains share a common fold, and they are arranged into tandems of two approximately symmetrical ZnF subunits which adopt a compact global form for the presence of an antiparallel β-sheet linker. However, no obvious rules for MBNL1 function or activity have been identified from the structure of its RNA binding domains [12,22,24].

**Figure 1:**
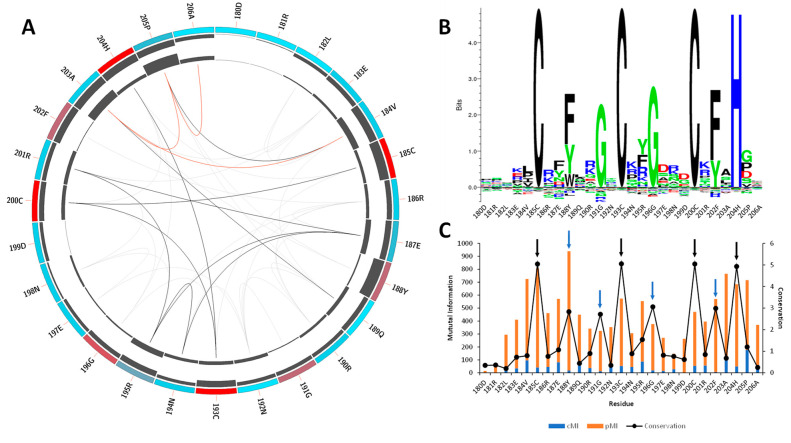
**Mutual information and conservation analysis of the CCCH domain protein family.** (**A**) Circos representation of the CCCH domain family. Square boxes indicate the KL conservation score (from red—highest values to cyan—lowest values). The cumulative mutual information (cMI) and proximity mutual information (pMI) scores are represented as histograms in the inner circle. Lines in the center connect pairs with a MI score higher than 6.5. Red lines represent the top 5%, black lines are between 95% and 70%, and gray lines indicate < 70%. (**B**) The KL sequence logo of selected nodes. (**C**) A histogram representing the conservation per residue, cMI and pMI , within 5 Å threshold. The black arrows represent the CCCH motif, and the blue arrows correspond to residues Phe188, Gly191, Gly196, and Phe202. The sequence id numbers correspond to those in the Circos representation.

According to sequence data retrieved from Pfam [25] in the moment of the analysis, the CCCH-motif core is highly conserved, especially among the MBNL proteins. Mutual information analysis was performed to identify residues involved in MBNL1-RNA contacts. The results showed that the most conserved amino acids are the constituents of the CCCH motif (red), followed by Phe188, Gly191, Gly196, and Phe202, which are located at a 2 and 3 amino acid distance to the Zn coordinated site (Figure 1A).

Tridimensional distance analysis showed that these residues are in close contact with the CCCH motif, which indicates a tightly conserved core. The KL logo shown in Figure 1B further confirmed the coevolution of the CCCH motif with the aforementioned residues. Figure 1C indicates that those residues located near the active sites (black arrows) exhibit coevolutionary trends, as stated by the cumulative mutual information (cMI) values. Notice that the proximity mutual information (pMI) values show increasing trends near the conserved residues Phe188, Gly191, Gly196, and Phe202 (blue arrows). In fact, the aromatic ring of Phe202 that is present in the ZnF3 of MBNL1 was observed to form stacking interactions with cytosines in the RNA structure and facilitate the macromolecular interaction [11,26]. The high coevolution propensity observed in this (F/Y)GG(F/Y) motif into the RNA binding site can also be inferred by examining the MI values, and further mobility analyses could reveal a correlation between coevolution and motions of this region. Particularly, inspection of the MBNL1 and MBNL2 sequences revealed that the (F/Y)GG(F/Y) motif is only present in MBNL1 ZnF3. On the contrary, the motif in ZnF1, ZnF2, and ZnF4 of MBNL1 is reduced to (F/Y)G(F/Y), as shown in Figures S1–S3.

Furthermore, we observed that residues Arg186, Arg190, Arg195, and Arg201 near the (F/Y)GG(F/Y) motif of ZnF3 also show high pMI values (Figure 1C), suggesting that a coevolutionary trend might exist. Liu et al. also observed that polar and charged residues presented high coevolution and high mobility, especially Ser, Asn, and Lys residues [27]. In line with that observation, conservation values observed in the KL logo (Figure 1B) show that Arg and Lys residues are the most frequent amino acids in such positions, which might indicate a relevant role for the recognition of the RNA backbone.

### 2.2. MBNL Domains from a Structural Perspective

**Figure 2:**
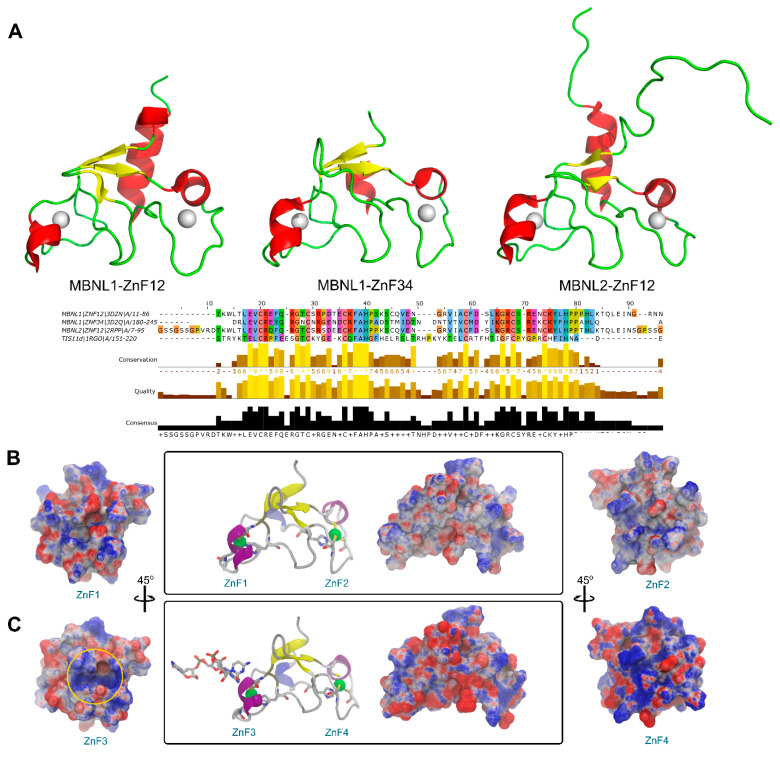
Sequence and structure comparison of MBNL1 ZnFs and MBNL2 ZnF1/2 domains. (**A**) Three-dimensional structure of MBNL1 ZnF1/2, MBNL1 ZnF3/4, and MBNL2 ZnF1/2, including sequence alignment, conservation, quality, and consensus sequence. (**B**) Electrostatic potential surface representation obtained using APBS contoured at ±10 kT/e for ZnF1/2 (PDB id 3D2N). (**C**) ZnF3/4 of MBNL1 binding an RNA fragment (PDB id 3D2S). The ZnF3 binding site region is contoured with a yellow circle.

Structural data is generally less abundant than sequence studies, and a greater difference is found in the MBNL protein family since very few experimental structures are available. A structural superposition of all MBNL1 ZnF domains and MBNL2 ZnF1/2 available evinced that the backbone conformation and topology are highly similar (Figure 2A). Nevertheless, information about the inter-domain linker is only partially available for the NMR ensemble of MBNL2 due to its high flexibility. On the contrary, the intra-domain linker between zinc finger pairs is not flexible and maintains a global compact fold. By doing so, both tandems may independently bind to different RNA regions. However, their binding affinities for RNA targets are different as pointed out by alanine substitution studies [28]. In fact, RNA binding and splicing activity is higher in a truncated MBNL1 protein that only contains a ZnF1/2 tandem rather than a truncated version with only ZnF3/4 [12]. Previous studies suggested that substrate recognition was assisted by coevolving residue pairs with enhanced global mobility that may improve its partner interactions with cognate binding motifs [27]. Thus, despite their high structural similarities, small changes in charge distribution, hydrogen-bonding potential, and flexibility behavior in each domain should yield differentiated binding and modify splicing capabilities.

An electrostatic potential analysis of both MBNL1 tandems (Figure 2B for ZnF1/2 and Figure 2C for ZnF3/4) was performed to highlight the differences between the binding interfaces of each ZnF domain. ZnF4 clearly exhibits the most positively charged surface due to an additional Lys residue at position 235. In total, five positive charges are present in this domain, which contrasts with the four positive charges present in ZnFs 1 to 3. The electrostatic surface on the ZnFs is mainly given by Arg and Lys residues. Due to the abundance and relative conservation of these amino acids, RNA binding is thought to occur first through interactions with not highly conserved residues which provide the necessary platform for the recognition of the RNA backbone and nucleotide bases with charged and π-stacking interactions. Then, the Phe202 aromatic ring that is present in the more conserved (F/Y)GG(F/Y) motif interacts with the RNA through stacking interactions, as reported in previous studies [11,26]. Additionally, Phe188 stacks with His204 from the CCCH motif and likely contributes to further stabilizing the core domain.

### 2.3. ZnF1/2 from MBNL1 and MBNL2 Exhibit Equivalent Large-Scale Motions

**Figure 3:**
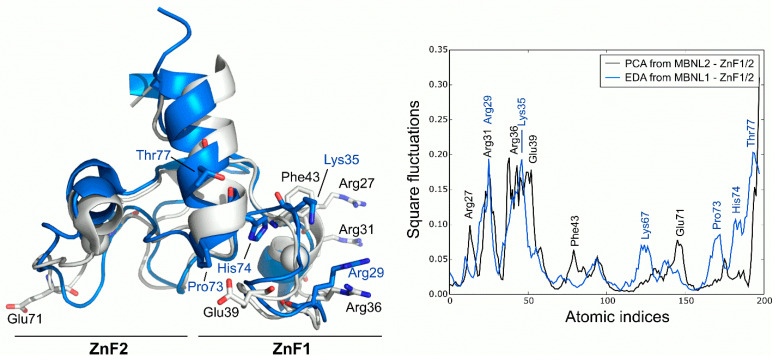
Global fluctuations of MBNL2 and MBNL1 ZnF1/2 computed from their backbone heavy atoms. Atomic indices correspond to residues 24 to 75 for MBNL1 (blue structure) and 26 to 77 for MBNL2 (white structure).

PCA analysis of the MBNL2 ZnF1/2 NMR ensemble revealed that the experimental global fluctuations are localized in charged and polar residues that are mainly characterized by Arg27, Arg31, Ser37, and Glu39 in the ZnF1 domain and Glu71 in the ZnF2 domain. These results are in line with the co-evolvability analysis, but the fact that the compactness of the protein is not dynamically conserved in both domains despite their high structural and sequence similarity is particularly interesting. The CCCH motifs present invariably low fluctuations, yet the high mobility of the conserved aromatic Phe43 residue is noteworthy due to its potential role in RNA binding. In fact, Edge et al. pointed out that alanine substitution in this position of ZnF1 of MBNL1 did affect its ability to activate splicing [12]. Computed MD fluctuations of the MBNL1 ZnF1/2 tandem were obtained using EDA and compared to the fluctuations of MBNL2 ZnF1/2. Interestingly, the PC1 of MBNL1 and MBNL2 yielded a modest correlation (Pearson correlation coefficient, r = 0.61), which suggests that both domains should have equivalent global low-frequency dynamics. Indeed, superposition of the fluctuations extracted from both tandems demonstrated that global fluctuations were localized into the same regions, following a similar trend (Figure 3). The inter-domain linker region of MBNL1 also exhibited high fluctuations due to the high mobility of its residues along the trajectory, especially on residues Pro73, His74, and Thr77. The highest fluctuations were observed in charged and polar residues next to the CCCH motif, as noticed before. These results show that, despite the large noise inherent to the technique, there is a good overlap in the behavior of AS control regions in both MBNL1 and MBNL2.

### 2.4. Intrinsic Fluctuations of ZnF3/4 Differ from Those of ZnF1/2

**Figure 4:**
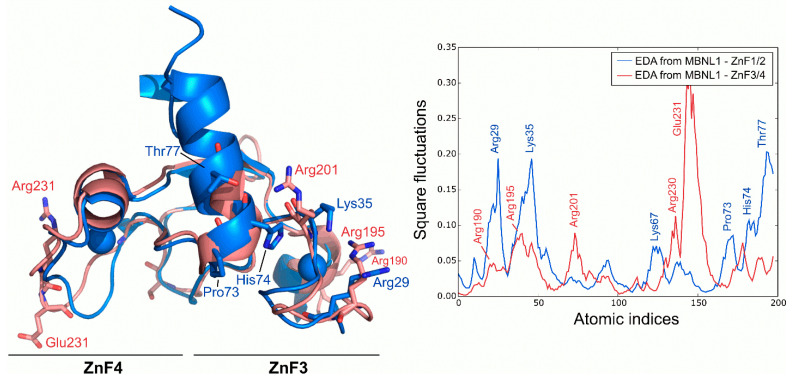
Global fluctuations of MBNL1, ZnF1/2, and ZnF3/4 computed from their backbone heavy atoms. Atomic indices correspond to residues 24 to 75 for ZnF1/2 (blue structure) and 185 to 236 for ZnF3/4 (red structure).

Following the dynamics analysis, a similar approach was applied to ZnF3/4 using MD trajectories. Although ZnF3/4 has a highly similar sequence and structure compared to ZnF1/2, ZnF3/4 dynamics strongly differ from those observed for ZnF1/2 (Figure 4). RMSD of the Cα between ZnFs after the simulation averaged 3.11 Å, but no structural rearrangements were observed in the protein’s core along the trajectory. Main deviations were located at the 310-helix element. ZnF3/4 presents its highest fluctuations onto the ZnF4 domain, which contrasts with the first tandem that exhibits higher fluctuations around the ZnF1 domain. Interestingly, global fluctuations from ZnF3/4 remarkably differ from those observed in ZnF1/2 (r = −0.01). An analysis of the essential subspace overlap between ZnF1/2 and ZnF3/4 further supported our results. The conformational coverage between both domains turned out to be low (27%), indicating that global motions are not conserved between both tandems.

### 2.5. Effect of RNA Binding over Local Fluctuations

**Figure 5:**
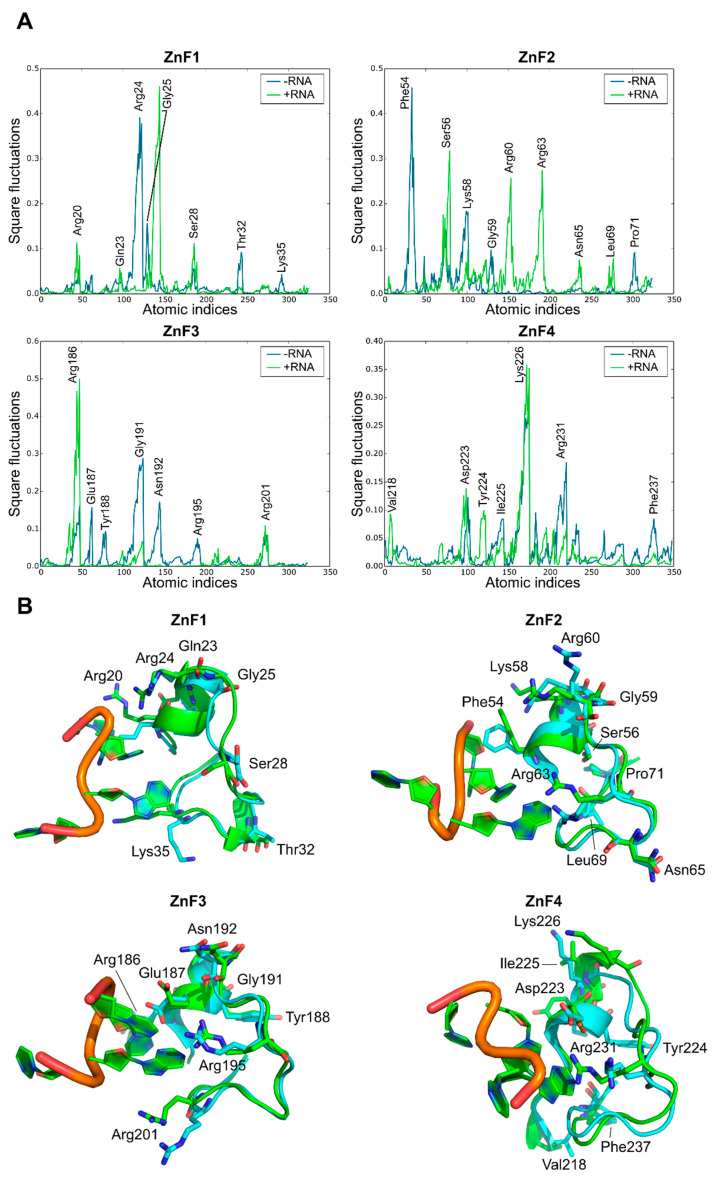
Effect of RNA binding on local fluctuations of MBNL1 ZnFs. (**A**) Local fluctuations extracted from the MD simulations for each domain alone (−RNA) or bonded to RNA (+RNA). Only the atomic indices of the RNA binding site are represented (ZnF1: 18 to 38; ZnF2: 52 to 72; ZnF3: 184 to 204; and ZnF4: 218 to 238). (**B**) Relevant fluctuating amino acids in an unbound (blue) and bound (green) state.

A comparative MD analysis between RNA-bound and unbound complexes was conducted to probe structural changes into the RNA binding sites of MBNL1. Local fluctuations were measured for each ZnF binding pocket in the presence or absence of the UGCU fragment. Figure 5A illustrates the main differences between each pair of trajectories. The local analysis and simulations indicated that, in general terms, most of the polar and charged residues fluctuations increase upon RNA binding (e.g., Ser56, Arg60, and Arg63 in ZnF2, Arg186 in Znf3, and Lys226 in ZnF4). On the contrary, Arg24 of ZnF1 fluctuations were suppressed due to anchoring to the RNA backbone through charge-based interactions (Figure 5B). The GC fragment strongly bonded to the binding pockets using concerted charged and π stacking interactions and hydrogen-bonding networks, which is in agreement with structural studies performed by Teplova et al. [11]. Interestingly, highly conserved Gly residues (specifically Gly25, Gly59, and Gly191) showed improved local mobility, especially in ZnF1 and ZnF3. These amino acids correspond to non-structured regions of the binding pocket adjacent to the α-helix. Visual inspection of MD trajectories showed that these binding regions alternate between ‘open’ and ‘closed’ configurations, accommodating the binding site and enhancing local interactions.

A significant correlation was found between RNA-bound and unbound ZnF4 local fluctuations (r = 0.76), meaning that the binding process does not induce significant changes into the ZnF4 pocket. Conversely, ZnF3 yielded a modest correlation between both configurations (r = 0.36), while ZnF1 and ZnF2 varied greatly between both states (r = 0.01 and r = 0.03 respectively). Indeed, binding and signaling effectivity could be partially explained by tight pocket packing and the restricted mobility of some residues in global modes upon RNA binding. In addition, high pMI values for these residues suggests a coevolutionary trend which may correlate with binding affinity. More interestingly, local fluctuations may not be only altered upon RNA binding to the ZnF domain, but also upon binding to the contiguous ZnF domain. For instance, ZnF1 local fluctuations were greatly modified when ZnF2 was in its bound or unbound configurations. The same phenomenon was observed for ZnF2 and ZnF3, but only ZnF4 can maintain its structural configuration when ZnF3 binds to the RNA (r = 0.87).

As a general trend, these analyses and simulations indicate that RNA may modify local fluctuations upon binding as well as modify structural protein couplings in neighboring binding pockets, except for ZnF4. The full correlation table can be found in Supplementary Material, Table S1.

### 2.6. Both ZnFs of MBNL1 Have Differentiated Affinity for RNA

**Figure 6:**
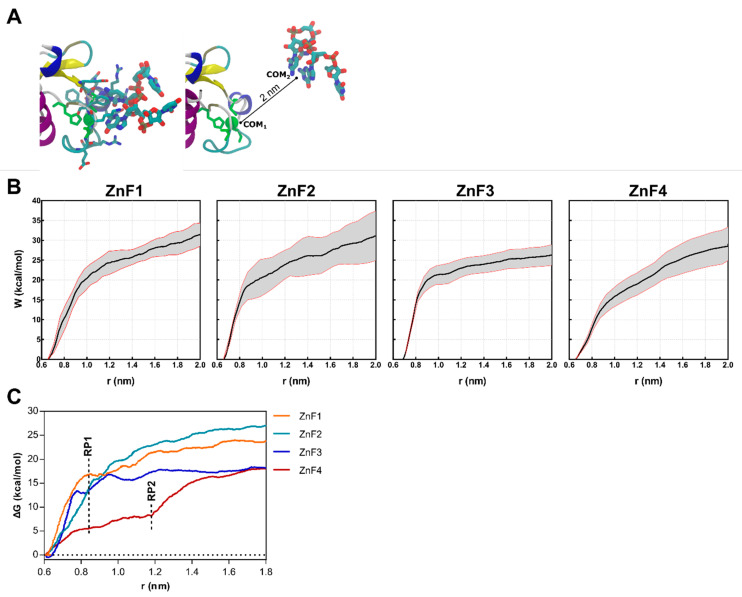
Results obtained from the sMD procedure on each of MBNL1’s ZnFs-RNA systems. (**A**) Representative snapshots of the initial (left) and final (right) coordinates of the sMD pulling process. The CCCH motif of each ZnF is colored green. (**B**) Cumulative work profiles for each ZnF vs. distance from the COM (r) between ZnF and RNA ligand. Black and red lines represent the mean and the 95% confidence interval. (**C**) Potential of Mean Force (PMF) for each ZnF extracted from the mean work profile. RP1 and RP2 correspond to the observed rupture points.

Steered molecular dynamics (sMD) were conducted on ZnF-RNA complexes to complete the picture of the impact of RNA binding over the structural rearrangements of MBNL1. Forces were applied to pull the UGCU fragment in order to describe the putative substrate binding/unbinding process for each domain (Figure 6A). The starting points for this study were 20 randomized frames from each ZnF-RNA complex equilibrated trajectory, which made a total of 80 pulling simulations. Each simulation computed the work of pulling the center of mass (COM) of the ZnF binding site and the GC pair 2 nm away from the initial coordinates. A spring constant of 556 nN·nm^−1^ and a constant velocity of 0.14 nm·ns^−1^ were used to compute the cumulative work profile for each system. Pulling force peaked at ~0.85 nm in all system models (Figure 6C), with a subsequent rearrangement in the adjoining GC binding pockets. No contribution of the U nucleobase to the binding/unbinding process was observed, except for the binding of Arg residues to the backbone of the first and fourth nucleotide.

As shown in Figure 6B, a statistically significant difference is observed between ZnF3 and the first and second domains (*p* < 0.01). ZnF1 and ZnF2 pulling simulations yielded very close work values (31.14 ± 5.03 and 31.43 ± 2.35 kcal·mol^−1^, respectively). Furthermore, ZnF3 (W = 26.29 ± 4.52 kcal·mol^−1^) clearly exhibited the lowest cumulative work, followed by ZnF4 (W = 29.10 ± 5.01 kcal·mol^−1^). This observation agrees with point mutation studies [24,29] that revealed that ZnF1/2 domains are required for an effective splicing on most targets.

The PMF (Figure 6C) was recovered from the successive pulling simulations using the Jarzynski equality [30]. As stated above, the rupture point (RP1) was located at ~0.85 nm when the RNA was completely out of the binding site and showed no interactions with ZnF. However, ZnF4 simulations consistently showed additional interactions at 0.85 nm which mainly involved Met222, Tyr224, and Lys226. Notice that Met222 and Tyr224 are only present in ZnF4, as shown in Figure 2A, and stabilize the RNA-protein complex until ~1.20 nm (RP2). In contrast with previous studies that hypothesized that MBNL1 ZnF4 could establish protein–protein interactions (PPI) with another MBNL1 ZnF4 unit through Tyr224, Gln244, and Tyr236 contacts, our simulations suggest that only Tyr224 mobility is suppressed upon RNA binding, which directly contributes to RNA stabilization, but no changes are apparent in Tyr236 and Gln244. Recent studies showed that mutation of these aromatic residues did not provide any effect in functional assays [10]. Nonetheless, cavities could be the sites for other PPI, and their composition and shape complementarity may be related to binding affinity and specificity.

**Figure 7:**
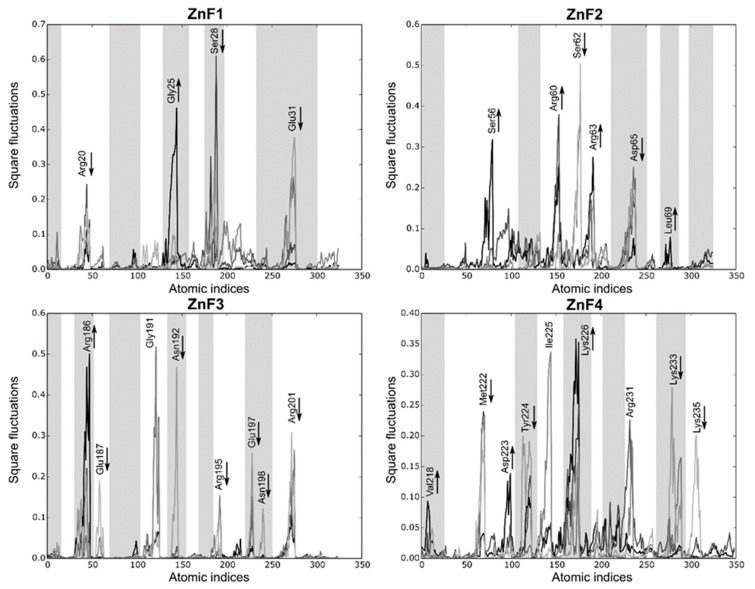
Local fluctuations extracted from the SMD simulations at four equally distributed segments of the trajectory. The binding process fluctuations (reversed SMD trajectory) are represented in light gray (unbound) to black lines (bound). Only the atomic indices of the RNA binding site are represented (ZnF1: 18 to 38; ZnF2: 52 to 72; ZnF3: 184 to 204; and ZnF4: 218 to 238). The most relevant fluctuations that increase or decrease during the binding process are indicated with an arrow. Grayed regions are not involved in the RNA-binding process.

Local fluctuations were computed at four equally distributed segments extracted from the sMD process to elucidate detailed differences between the binding domains (each segment represents 2 ns). Figure 7 shows that local fluctuations are more pronounced in ZnF2 and ZnF4, while the other domains yield more localized fluctuations. Arg, Lys, Glu, and Asp mainly represent the pocket mobility and, not surprisingly, most of them reduced upon binding. Interestingly, Met222 and Tyr224 interactions in ZnF4 are suppressed during the binding process, showing that their interactions upon RNA binding are remarkably stable. Notice that half of the fluctuating residues (marked as greyed regions in Figure 7) are not at a binding distance to the RNA target. Visual inspection of the sMD trajectories from ZnF1 and ZnF3 do not provide any evidence about the binding process differences, and the Cα RMSD between them is sustained around 1.4 Å. However, this fact can be reconciled if both local fluctuations and pocket rearrangements are considered. We noticed that residues Gly25 and Gly191 exerted dynamic control of the binding region, which induced subtle changes in protein dynamics. General differences between the binding pockets were observed in terms of total volume distribution. Binding pockets of ZnF2 and ZnF4 exhibited a broad volume distribution that agreed with the major number of local fluctuations into these regions. Mean pocket volume of ZnF2 and ZnF4 distributions were close (57.7 Å^3^ and 53.3 Å^3^, respectively), and their distribution ranged from 12 Å^3^ to 172 Å^3^. This effect indicates both local and large rearrangement of the nearby pockets upon RNA binding. On the contrary, ZnF1 and ZnF3 displayed similar distributions whose statistical mean was located at 39.4 Å^3^ and 32.5 Å^3^, respectively. Volume distribution plots for each ZnF are included in Supplementary Material Figure S4.

## 3. Materials and Methods

### 3.1. Conservation and Coevolution Analyses

The multiple sequence alignment was conducted using data from Pfam [25] (PF00642, containing Zinc finger CX8CX5CX3H type and similar). Mutual information (MI) analysis between amino acids at the ith and jth positions were computed with MISTIC [31]. This server uses an APC corrected MI to reduce background mutual information and translate them into Z-scores. The cumulative MI (cMI) and proximity MI (pMI) were computed for each residue with a Z-score threshold > 6.5, as previously described.

### 3.2. System Preparation

Structural models of ZnF1/2 and ZnF3/4 in a free state were retrieved from the Protein Data Bank (PDB ids 3D2N and 3D2Q, respectively) [9]. The NMR structure of ZnF1/2 from MBNL2 correspond to the PDB id 2RPP [32]. Residues 245 to 253 in the C-terminal region of ZnF3/4 were homology modeled using ZnF1/2 as a model template in order to extend the C-terminal region. A ZnF3/4-r(CGCUGUG) system was retrieved from the PDB (3D2S) and prepared as follows: the RNA model bound to ZnF3 was reduced to a 4-nt long sequence and mutated to UGCU (corresponding to a YGCY motif present in CUG repeats). Then, ZnF1, ZnF2, and ZnF4 bound to the RNA fragment were prepared by sequentially aligning the binding regions with the ZnF3 template model.

### 3.3. Molecular Dynamics

Short MD were run in order to guarantee the stability of the modeled systems. Each system was neutralized using the tLeap module in AMBER14 [33] with Cl- ions and solvated with TIP3P water molecules in a 12 Å truncated octahedral box. The Amber ff12SB force field was used in all the simulations [33]. Zinc atoms were treated according to the cationic dummy atom approach using Pang et al.’s all-atom force field parameters [34]. Each system was minimized using a conventional two-stage process. First, all residues except solvent were restrained with a 100 kcal·mol^−1^·Å^−2^ restraint force and minimized with 2500 steepest descent steps, followed by 2500 conjugate gradient steps. A second minimization stage was performed without positional restraints using 10,000 steepest descent steps and 10,000 conjugate gradient steps. Each system was slowly heated to 300 K for 150 ps while constraining the solute with a force gradient of 8.0 to 0 kcal·mol^−1^·Å^−2^. Langevin dynamics with a collision frequency of 1 ps^−1^ were used. A 20 ps of pressure equilibration stage was then applied with isotropic scaling at 1 atm. A 100 ps pre-production stage was carried out using NVT ensemble and chemical bonds involving hydrogen atoms that were constrained using the SHAKE algorithm [35] which allowed an integration step of 2 fs in the production runs. Particle Mesh Ewald (PME) [36,37] was used in all calculations with a 9 Å long-range cutoff. The resulting models were used as the starting points for the sMD simulations.

### 3.4. Essential Dynamics and Principal Components Analysis

Essential dynamics analysis (EDA) and principal component analysis (PCA) were used to study the dynamic properties of experimental structures. PCA was completed by decomposing the covariance matrix, as previously described [38,39]. Likewise, PCs from MD trajectories were extracted using essential dynamics analysis (EDA). EDA modes were obtained by decomposing the covariance matrix for 30,000 equally distributed snapshots extracted from each simulation. Local and global fluctuations were computed from the PC1 extracted from either PCA or EDA. Global fluctuation analyses were based only on the Cα, N, and C backbone atoms of the model system. Overlap between EDA and PCA modes was calculated using the dot product of the corresponding eigenvectors. All PCA and EDA analyses were completed with ProDy 1.14 software [39]. The cpptraj module of Amber 14 [33] was used for root–mean–square deviation (RMSD) and to process the MD trajectories. The output was analyzed with VMD [40].

### 3.5. Steered Molecular Dynamics

Four independent sMD experiments were performed to describe the binding/unbinding MBNL1-RNA process, pulling away the center of mass (COM) of the GC binding pair (considering all non-hydrogen atoms) from the COM of each binding pocket (residues at 4.5 Å from the RNA sequence). sMD simulations were performed with the same protocol described for the equilibration step previously described for cMD and initiated with the final structure obtained from the equilibration step. The pulling force was applied to the COMs from ~0.65 nm until a separation of 2 nm was achieved. A total of 20 successive sMD pulling experiments per complex were conducted with a constant velocity of 0.14 nm·ns^−1^ and a spring constant of 556 nN·nm^−1^. The total cumulative time for the sMD experiments was 800 ns. The potential of mean force (PMF) was reconstructed from the successive sMD simulations using the Jarzynski equation [30].

(1) 〈exp(−βW)〉=exp(−βΔG)

### 3.6. Pocket Analysis

MBNL1 binding regions were analyzed using POVME 2.0 [41]. The COM of the protein cavity was defined as described in the sMD (see Supporting Information), and inclusion regions were defined using a text based POVME input file. Grid spacing of 1.0 Å was used to create a field of equidistant points, and the volume was measured using the POVME 2.0 plugin.

## 4. Conclusions

The MBNL protein family plays a prominent role in the regulation of alternative splicing (AS) during development, and its loss leads to a major pathological event known as the neuromuscular disease myotonic dystrophy (DM). Studies revealed that although the four zinc-finger domains of MBNL1 recognize the consensus motif YGCY RNA element, no obvious rules for MBNL1 function or activity have been identified so far. In fact, many aspects of protein function, such as cognate RNA binding, can be fully understood only in terms of an equilibrium ensemble of alternative structures, rather than a single static structure [22,24].

In this work we characterized the motions of the four zinc-fingers present in MBNL1 and provided a complementary picture to the experimental observations from a structural point of view using conventional and steered molecular dynamics. Sequence coevolution analysis of the CCCH domains of MBNL1 showed high fluctuations that determine the affinity in each ZnF domain and confirmed that coevolutionary trends affect function and protein dynamics. Combined coevolution propensity and conformational mobility on MBNL1 ZnFs suggest that charged and polar amino acids (especially Lys and Arg) are greatly involved in substrate recognition. Thus, these residues are also specific and flexible enough to mediate substrate selectivity.

Global and local fluctuations have been analyzed since the four ZnFs in MBNL1 have different RNA-binding and splicing activities. The simulations confirmed that conserved Gly residues adjoining to the α-helix enhance local mobility of the RNA-binding region. We also found that global fluctuations are remarkably different in each RNAbinding domain. On the one hand, the ZnF1/2 tandem structural configuration is conserved upon RNA binding, but local and global fluctuations are remarkably modified. On the other hand, the ZnF3/4 tandem is more resilient to changes in local motions. ZnF3 fluctuations show a modest correlation with those observed upon RNA binding. However, RNA-binding to ZnF4 produces a remarkable change into ZnF3 local motions. An intriguing finding is that our simulations showed that ZnF4 motions remain invariable during all possible events (free state, RNA-bound, and RNA-bound to its partner, ZnF3). Our simulations suggest that only Tyr224 mobility is suppressed upon RNA binding, directly contributing to RNA stabilization, but no other remarkable changes are apparent. Interestingly, ZnF4 has a noteworthy positive charge compared to the other ZnF domains, and the observed conformational adaptation of its pocket suggests an important role in early RNA binding recognition as well as favorable inter-domain binding capabilities.

To sum up this study, it can be concluded that the four ZnF domains provide distinct RNA-binding platforms in terms of structural sampling and mobility that may have implications for the differentiated splicing events observed in the literature. Although these results are in good agreement with experimental data, they are not conclusive, and further research would be required to validate these observations. However, to the best of our knowledge, the experimental assessment of the correlation between molecular mobility (local or global) and the functional activity of the protein remains challenging. The effect of the residues pointed out in the discussion on the RNA binding would be experimentally assessed, even in an indirect way (e.g., binding assays or directed mutagenesis). An important future line of work would also be to identify the contributions of non-structured regions, such as the inter-domain linker.

## Data Availability

The data presented in this study are available on request from the corresponding author.

## Supplementary Materials

The following supporting information can be downloaded at: https://www.mdpi.com/article/10.3390/ijms232416147/s1.
