# Supplementary material for: Cognate RNA-Binding Modes by the Alternative-Splicing Regulator MBNL1 Inferred from Molecular Dynamics
Source: Int J Mol Sci. 2022 Dec 18;23(24):16147. doi: 10.3390/ijms232416147 (PMC9780971; doi:10.3390/ijms232416147)
Supplement: Supplementary file 1 [file ijms-23-16147-s001.zip › ijms-2044354-supplementary.pdf]

Supporting Information

Cognate RNA-Binding Modes by the Alternative-Splicing Regulator MBNL1 Inferred from Molecular Dynamics

Àlex L. González, Daniel Fernández-Remacha, José Ignacio Borrell, Jordi Teixidó, Roger Estrada-Tejedor*

Grup de Química Farmacèutica (GQF), IQS School of Engineering, Universitat Ramon Llull, Barcelona, Spain

* Correspondence: roger.estrada@iqs.url.edu

Contents

Table S1. Pearson correlation coefficient between RNA-bound and unbound local fluctuations for each ZnF domain. 2

Figure S1. Mutual information and conservation analysis of the CCCH domain in MBNL1 ZnF1 protein family. 3

Figure S2. Mutual information and conservation analysis of the CCCH domain in MBNL1 ZnF2 protein family. 4

Figure S3. Mutual information and conservation analysis of the CCCH domain in MBNL1 ZnF4 protein family. 5

Figure S4. Volume of the pocket computed during the sMD process for each ZnF 6

Table S1. Pearson correlation coefficient between RNA-bound and unbound local fluctuations for each ZnF domain. Tandem partner refers to each ZnF-partner into the same tandem (ZnF1 partners ZnF2, and ZnF3 partners ZnF4).

	**ZnF1**	**ZnF2**	**ZnF3**	**ZnF4**
**ZnF1 - RNA**	0.01	0.05	**0.31**	0.12
**ZnF2 - RNA**	0.06	0.03	0.21	-0.06
**ZnF3 - RNA**	0.12	0.04	**0.36**	-0.09
**ZnF4 - RNA**	0.13	-0.04	0.08	**0.76**
**Tandem partner - RNA**	0.05	0.11	0.06	**0.87**

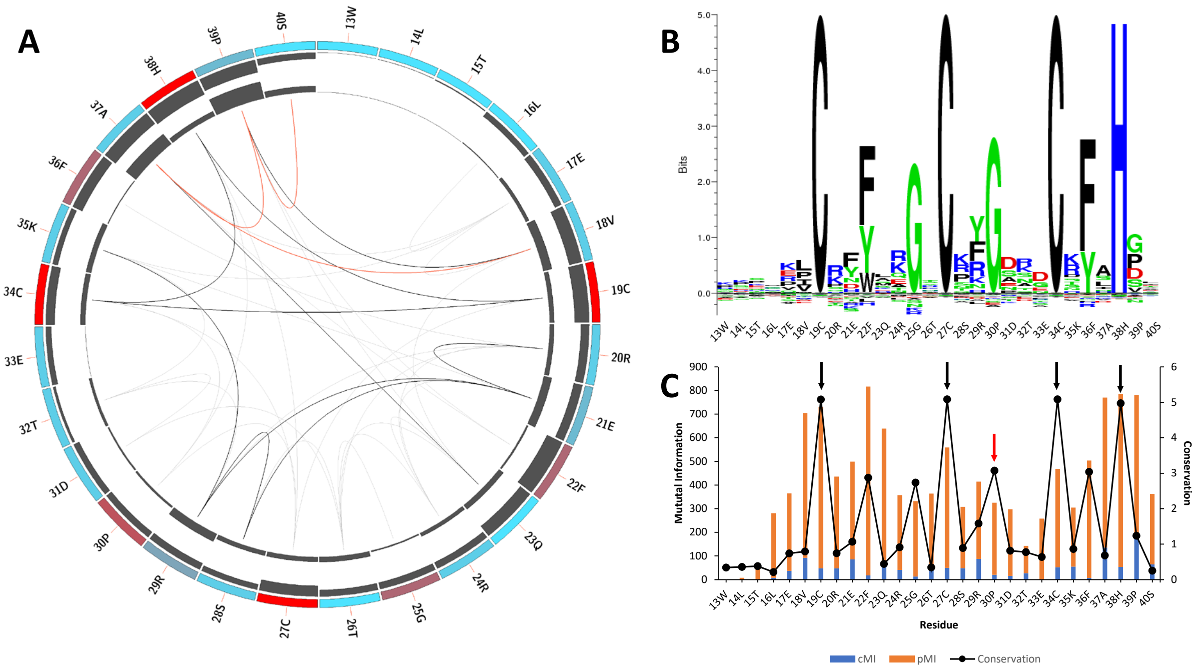

Figure S1. **Mutual information and conservation analysis of the CCCH domain in MBNL1 ZnF1 protein family.** (A) Circos representation of the CCCH domain family in ZnF1. Square boxes indicate the KL conservation score (from red-highest to cyan-lowest values). The cumulative mutual information (cMI) and proximity mutual information (pMI) scores are represented as histograms in the inner circle. Lines in the center connect pairs with a MI score higher than 6.5. Red lines represent the top 5%, black lines are between 95% and 70%, and gray lines indicate < 70%. (B) KL sequence logo of selected nodes. The second conserved Gly of the (F/Y)GG(F/Y) is noticeable for the alignment of ZnF1 Pro30 in the Multiple Sequence Alignment (MSA) from Pfam. Nevertheless, ZnF1 presents the (F/Y)G(F/Y) motif. (C) Histogram representing the conservation per residue, cMI and pMI within 5 Å threshold. Black arrows represent the CCCH motif and Pro30 is indicated in red. The sequence id numbers correspond to those in the circos representation.

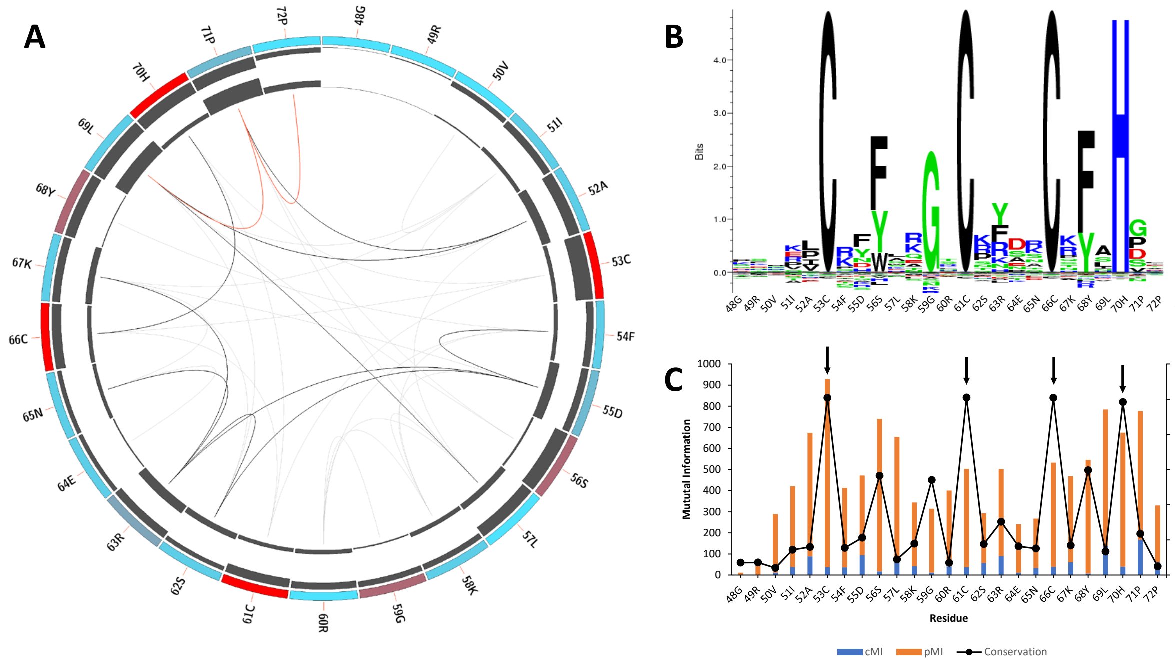

Figure S2. **Mutual information and conservation analysis of the CCCH domain in MBNL1 ZnF2 protein family.** (A) Circos representation of the CCCH domain family in ZnF2. Square boxes indicate the KL conservation score (from red-highest to cyan-lowest values). The cumulative mutual information (cMI) and proximity mutual information (pMI) scores are represented as histograms in the inner circle. Lines in the center connect pairs with a MI score higher than 6.5. Red lines represent the top 5%, black lines are between 95% and 70%, and gray lines indicate < 70%. (B) KL sequence logo of selected nodes. (C) Histogram representing the conservation per residue, cMI and pMI within 5 Å threshold. Black arrows represent the CCCH motif. The sequence id numbers correspond to those in the circos representation.

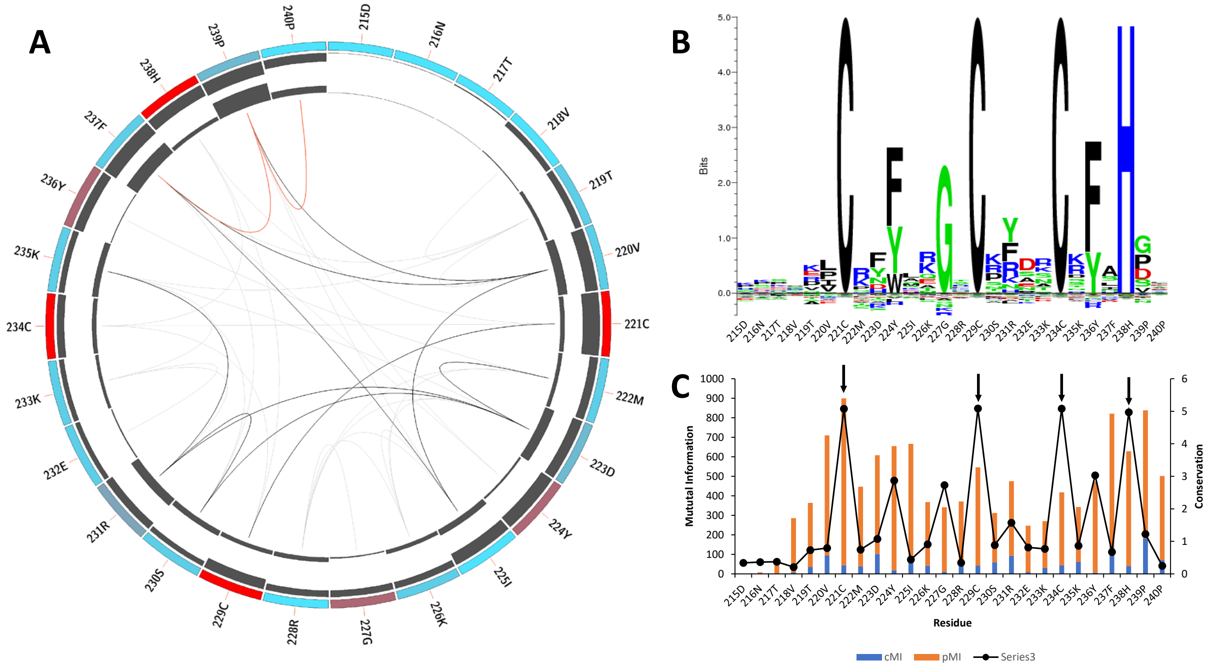

Figure S3. **Mutual information and conservation analysis of the CCCH domain in MBNL1 ZnF4 protein family.** (A) Circos representation of the CCCH domain family in ZnF4. Square boxes indicate the KL conservation score (from red-highest to cyan-lowest values). The cumulative mutual information (cMI) and proximity mutual information (pMI) scores are represented as histograms in the inner circle. Lines in the center connect pairs with a MI score higher than 6.5. Red lines represent the top 5%, black lines are between 95% and 70%, and gray lines indicate < 70%. (B) KL sequence logo of selected nodes. (C) Histogram representing the conservation per residue, cMI and pMI within 5 Å threshold. Black arrows represent the CCCH motif. The sequence id numbers correspond to those in the circos representation.

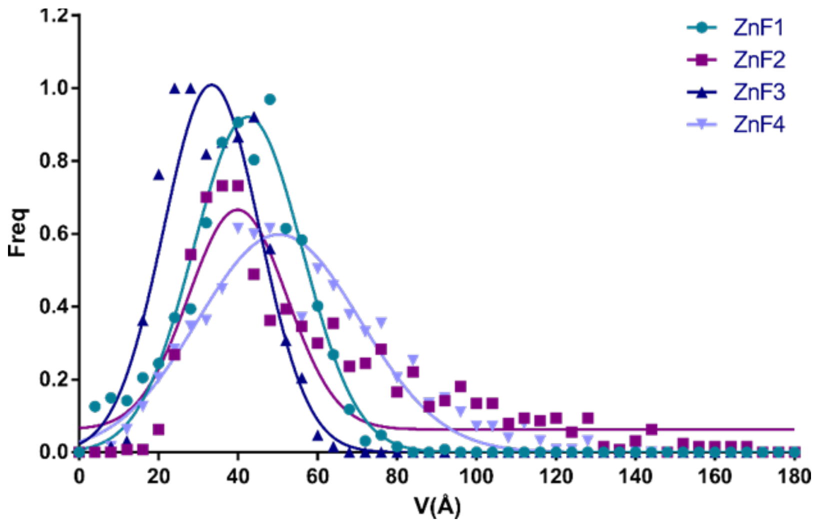

Figure S4. Volume of the pocket computed during the sMD process for each ZnF. Data has been smoothened using Gaussian distributions.
